# The impact of wearable cardioverter-defibrillator use on long-term decision for implantation of a cardioverter-defibrillator in a semirural acute care hospital

**DOI:** 10.1007/s10840-020-00898-5

**Published:** 2020-11-17

**Authors:** Anil-Martin Sinha, Jens Bense, Wolfgang Hohenforst-Schmidt

**Affiliations:** 1 Department of Cardiology, Sana Klinikum Hof, Eppenreuther Str. 9, 95032 Hof, Germany; 2 Department of Cardiology, Teaching Hospital of the Friedrich Alexander University Erlangen/Nürnberg, Erlangen/Nürnberg, Germany

**Keywords:** Arrhythmia, Cardiomyopathy, Implantable cardioverter-defibrillator, Sudden cardiac arrest, Sudden cardiac death, Wearable cardioverter-defibrillator

## Abstract

**Purpose:**

Large-scale multi-center studies have reported on efficacy of the wearable cardioverter-defibrillator (WCD). However, outcomes focused on WCD patients treated at community-based acute care centers are lacking.

**Methods:**

Patients with cardiomyopathy were included when left ventricular ejection fraction (LVEF) at baseline was ≤ 35%. There were 120 patients meeting the criteria who also had LVEF measured at baseline and after 90 days of WCD use.

**Results:**

After 90 days of WCD use, there were 44 (37%) patients in whom LVEF improved to > 35%. Comparison of patients, by whether LVEF improved or not, indicated that median days of WCD wear and hours of daily use were similar as well as characteristics, such as gender, age, and starting LVEF; and diagnoses leading to WCD prescription were similar between groups as were symptom-based prescription of medications. At the end of WCD use, improved LVEF > 35% correlated with fewer implantable cardioverter-defibrillator (ICD) implants. There were 4 (3%) episodes of new atrial fibrillation detected during WCD use. The WCD appropriately delivered a shock to 3 (2.5%) patients with VT/VF being terminated by the first shock. All shocked patients survived for at least 24 h post-shock.

**Conclusions:**

During WCD use, ischemic and non-ischemic cardiomyopathy patients manifest improved LVEF by 90 days. Long-term care decisions, such as implantation of an ICD, were influenced by LVEF improvement and occurrence of spontaneous VT/VF. The WCD protected patients from sudden cardiac death (SCD) until patient response to guideline-directed medical therapy could be determined.

## Introduction

Outside the hospital, acute care is dependent on access to emergency medical services (EMS). Patients who reside in semirural settings have inherently longer EMS response times [1, 2]. Following onset of ventricular tachycardia (VT) or ventricular fibrillation (VF), the likelihood of successful resuscitation decreases at a rate of approximately 10% per minute of delayed defibrillation [3]. However, automated defibrillators placed in the home have been unsuccessful when measured against improved survival following VT/VF [4].

Although the Vest Prevention of Early Sudden Death Trial (VEST) failed to achieve statistical significance for sudden cardiac death (SCD) reduction at 90 days [5], multi-center registries and prospective studies have provided convincing evidence that patients benefit from wearable cardioverter-defibrillator (WCD) use [6, 7]. Feldman et al. found in 289 patients at high risk for sudden death that a WCD was beneficial in detecting and effectively treating ventricular tachyarrhythmias in patients at high risk for sudden death who were not clear candidates for an implantable cardioverter-defibrillator (ICD) [8]. Moreover, the WCD seemed to be useful as a bridge to transplantation or ICD in selected patients [8].

As the risk for sudden cardiac arrest during the waiting period before ICD implantation was unclear, Epstein et al. analyzed detected arrhythmias and shocks delivered in 853 WCD patients with reduced left ventricular ejection fraction (LVEF) during the first 3 months post-myocardial infarction (MI) [9]. They found that 1.4% of patients were successfully shocked within the first 3 months with a resuscitation survival rate of 91%. Furthermore, sudden cardiac death risk in post-MI patients was highest in the first month of WCD use [9]. Chung et al. assessed patient compliance and effectiveness of antiarrhythmic treatment by WCD in 3569 patients [10]. They reported that compliance was satisfactory with 90% wear time in 50% of patients and low sudden death mortality during use, and that survival was comparable to that of ICD patients. [10].

However, there are few reports about WCD experience when patients are discharged from community-based hospitals. The current study prospectively enrolled patients with increased risk of VT/VF indicated by cardiomyopathy and impaired LVEF, and presents the WCD experience from a single-center acute care hospital serving a semirural region.

## Methods

### Consent

Before the start of WCD wear, all patients are consented to use their data for quality monitoring, healthcare operations, and research purposes.

### Cohort

The prospective analysis included patients prescribed the WCD at the Sana Klinikum Hof, Germany, from February 2012 to May 2017. Prescription criteria were based upon clinical characteristics at hospital admission which were LVEF ≤ 35% and either ischemic (*n* = 46, 38%; acute myocardial infarction or ischemic cardiomyopathy), non-ischemic (*n* = 69, 58%; myocarditis, congestive heart failure, or dilated cardiomyopathy), or others (*n* = 5, 3%; ICD infection leading to explant or genetic cardiomyopathy). Patients were enrolled if they were prescribed the WCD, presented with LVEF ≤ 35% and cardiomyopathy, and had LVEF measured after 90 days of WCD use. LVEF was measured using Simpson’s biplane method.

### Patient characteristics

Patient characteristics and medical treatment were evaluated at the beginning and after 90 days of WCD use.

### Wearable cardioverter-defibrillator use

Patient use data were obtained from the commercial database. Days of wear was the sum of days in which the WCD was worn for greater than 15 min. Hours of daily use was the ratio of the sum of hours and the sum of days minus 1. The purpose of the 1-day adjustment was to correct for the partial days available on the first and last days of prescribed wear.

### Arrhythmia data analysis

Electrocardiogram (ECG) data recorded by the WCD system were remotely transmitted, screened for whether they included information on heart rate and rhythm, and analyzed by the authors, including 2 cardiologists. The WCD recorded the automated detection of and delivered shock in response to VT and VF events. The WCD includes a 2-lead ECG monitoring system and continuously monitors the patient for heart rate and rhythm. In the event of VT or VF, the WCD will record the ECG immediately before and after delivering a shock. Signals other than VT/VF can also initiate an ECG recording. These include irregular and fast heart rhythms that are of supraventricular origin. The lengths of recording typically vary between 45 s and several minutes depending of the nature of the detected heart rhythm and the patient’s interaction with the device’s response buttons. VT or VF was ventricular tachyarrhythmia lasting longer than 30 s. VT was defined as having monomorphic or polymorphic characteristics, and VF as an inconsistent morphology. A single event included all ECG records of VT or VF occurring within 24 h of the index arrhythmia. Appropriate shocks were defined as being delivered in response to an episode of VT or VF. Inappropriate shocks were WCD shocks delivered in the absence of VT or VF.

### Post-shock follow-up

Data associated with shock events include the ECG record encompassing the event, the number of shocks administered, and whether shock resulted in conversion. All shock events were investigated by the responsible physician. Outcomes were tabulated, with survival defined as alive 24 h after receiving a shock.

### Statistical analysis

Statistical analysis was performed using R [11]. Data were reported as the median and interquartile range (IQR) or as the number of patients and percentage of total patients. Categorical data were analyzed using Fisher’s exact test and continuous data by the Wilcoxon test.

## Results

### Patient selection and characteristics

**Fig. 1:**
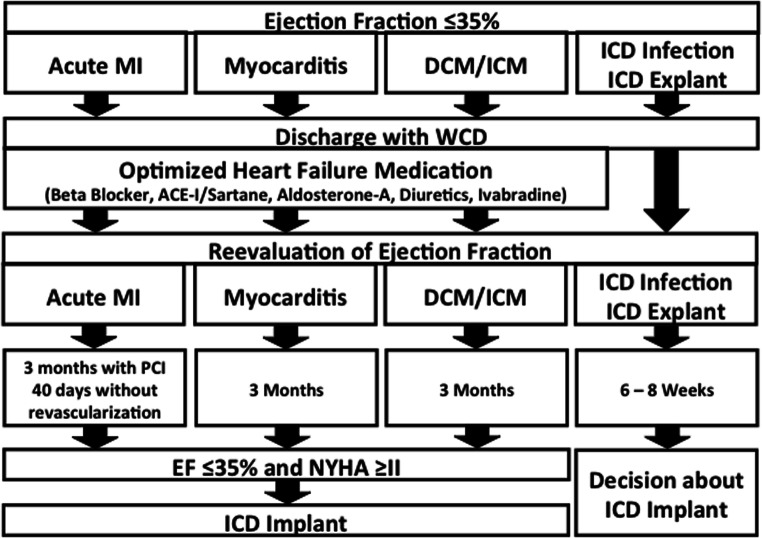
Sana Klinikum Hof, Germany, algorithm for prescribing the wearable cardioverter-defibrillator (MI, myocardial infarction; DCM/ ICM, dilative cardiomyopathy/ischemic cardiomyopathy; ICD, implantable cardioverter-defibrillator; ACE-I, angiotensin converting enzyme inhibitor)

WCD prescription was based upon standard clinical algorithm performed at the Sana Klinikum in Hof, Germany (Fig. 1). Eligible patients diagnosed with acute MI were prescribed the WCD for 3 months when percutaneous coronary intervention (PCI) was performed or for 40 days without revascularization. Prescription of the WCD was for 3 months when the diagnosis was non-ischemic, non-acute ischemic, or other eligible cardiomyopathy. At the end of WCD prescription, LVEF was reevaluated and decision to implant an ICD followed European Society of Cardiology (ESC) guidelines.

Baseline patient characteristics are summarized in Table 1. The 120 patients were primarily male (79%) with median age 66 years. Cardiomyopathy leading to WCD prescription were primarily ischemic and non-ischemic (38% versus 58%) with reduced LVEF ≤ 35%. All patients received optimized medication for heart failure, including beta blocker (92%), ACE inhibitor (80%), and diuretic (86%).

**Table 1:** Wearable cardioverter-defibrillator patient characteristics

Characteristic	Included, *N* = 120	90 day LVEF ≤ 35%, *N* = 76	90 day LVEF > 35%, *N* = 44	*p* value
Demographics				
Male, *N* (%)	95 (79)	61 (80)	34 (77)	0.82
Age, median (IQR)	66 (56, 75)	69 (54, 74)	64 (54, 74)	0.11
LVEF at start, median (IQR)	26 (20, 30)	26 (22, 30)	26 (22, 30)	0.98
Reason for WCD				
Ischemic, *N* (%)	46 (38)	29 (38)	17 (39)	1
Non-Ischemic, *N* (%)	69 (58)	44 (58)	25 (57)	1
Others, *N* (%)	5 (4)	3 (4)	2 (5)	1
Medications				
Beta blocker, *N* (%)	111 (92)	70 (92)	41 (93)	1
ACE inhibitor, *N* (%)	96 (80)	61 (80)	35 (80)	1
Diuretic, *N* (%)	103 (86)	64 (84)	39 (89)	0.59
Digitalis, *N* (%)	2 (2)	1 (1)	1 (2)	1
WCD use				
Days of wear, median (IQR)	48 (37, 62)	52 (40, 66)	45 (36, 56)	0.14
Hours daily use, median (IQR)	22.9 (21.2, 23.4)	23.0 (21.4, 23.4)	22.7 (20.1, 23.3)	0.30
ICD implanted at 90 days				
Received ICD, *N* (%)	71 (59)	68 (89)	3 (7)	< 0.001
No ICD, *N* (%)	44 (37)	3 (4)	41 (93)	< 0.001
Unknown, *N* (%)	5 (4)	5 (7)	0 (0)	0.16

*ICD*, implantable cardioverter-defibrillator; *IQR*, interquartile range; *N*, number; *WCD*, wearable cardioverter-defibrillator

### Patient characteristics after 90 days

Patient characteristics after 90 days of WCD use, distributed by whether LVEF surpassed 35% or not, are also summarized in Table 1. The distribution of gender (80% versus 77% male), median age (69 versus 64 years), and starting LVEF (26% versus 26%) was similar between both groups. The percentage of patients who achieved LVEF > 35% at 90 days was also similar for ischemic, non-ischemic, and other cardiomyopathy diagnoses. Symptom-based prescription of medication (e.g., beta-blockers, ACE inhibitors, diuretics and digitalis) was similar when compared by LVEF at follow-up. Comparison of WCD use by patients in these two groups indicated that median days of wear (52 versus 45 days) and hours of daily use (23.0 versus 22.7 h, an 18 min difference) were also similar. Overall, the WCD was worn by all patients for at least 3 days, and 112 (93%), 94 (78%), 33 (51%), and 11 (9%) patients wore the WCD for at least 7, 30, 60, and 90 days, respectively. Other than WCD delivered shock preceding an ICD implant, reasons for discontinuing WCD use prior to 90 days were not recorded during the study period.

### LVEF after 90 days follow-up

**Fig. 2:**
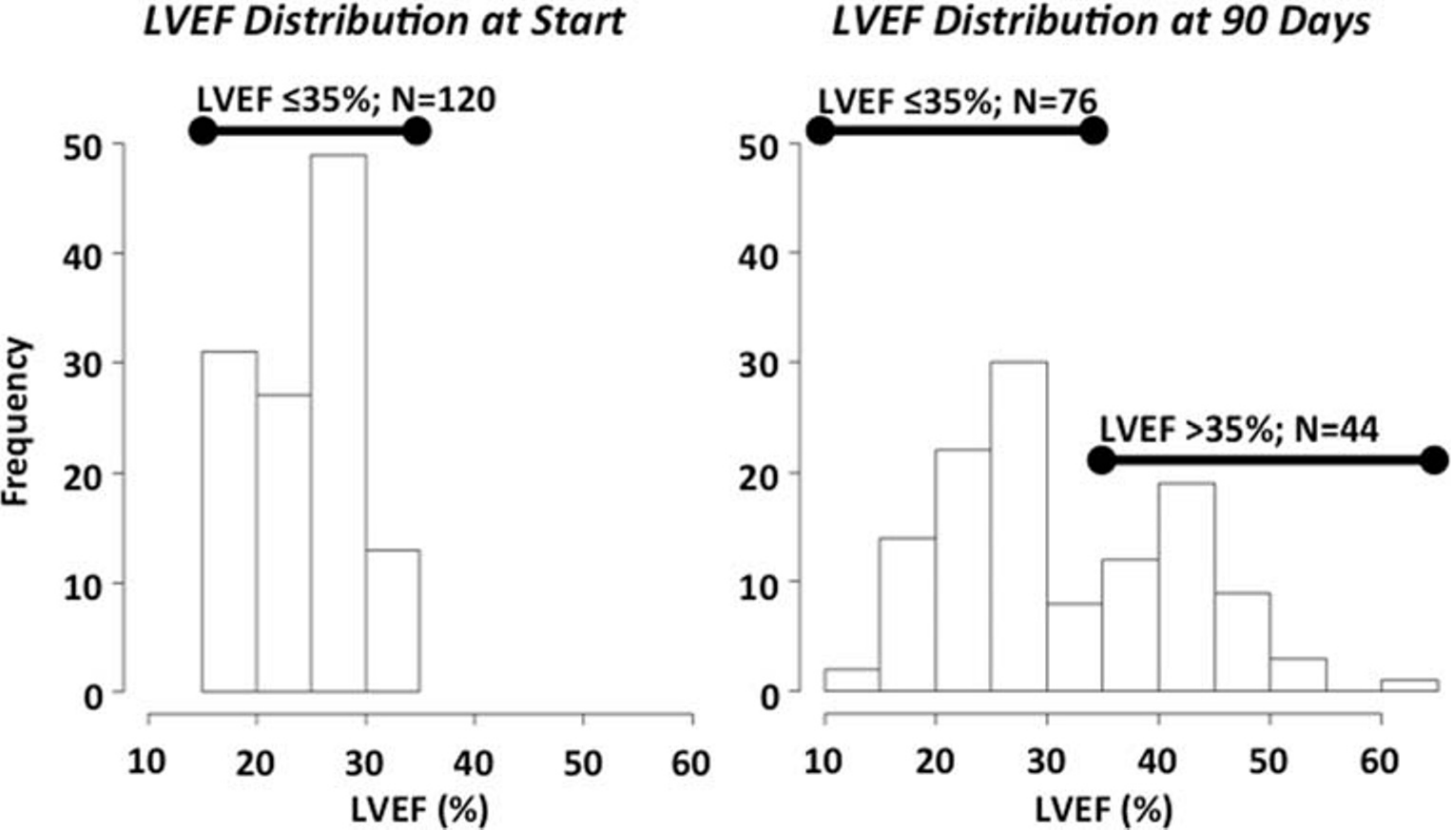
Histogram of patient left ventricular ejection fraction (LVEF) before (left panel) and after 90 days of wearable cardioverter-defibrillator use (right panel)

At the start of WCD wear, all patients had LVEF ≤ 35% with LVEF 25 to 30% being the most frequent, accounting for 49 (41%) patients (Fig. 2, left panel). After 90 days of WCD wear, there were 76 (63%) patients with LVEF ≤ 35% remaining while 44 (37%) responded to guideline-directed medical therapy (GDMT) as indicated by improved LVEF > 35% (Fig. 2, right panel). Analysis of patients by starting LVEF indicated that patients with severely reduced LVEF ≤ 25% were as likely to improve as were patients with LVEF above 25%. Thus, after 90 days of WCD wear, 22 out of 58 patients with LVEF ≤ 25% improved to LVEF > 35%, while 22 out of 62 patients with LVEF > 25% improved to LVEF > 35%, equivalent to one-third of patients.

### Appropriate wearable cardioverter-defibrillator therapy

Three patients (2.5%) ages 60, 78, and 67 years were shocked for VT/VF, 1 non-ischemic, and 2 ischemic (Table 2). In each patient, a single episode of VT/VF was detected on days of WCD wear 36, 6, and 17, and terminated by the first shock. No patient was shocked more than once and no patient was shocked inappropriately by the WCD or died while wearing the WCD. Survival was documented at least 24 h post-shock. The shocked patients were successfully implanted with an ICD for secondary prevention of SCD.

**Table 2:** Wearable cardioverter-defibrillator shocks

	Patient 1	Patient 2	Patient 3
Demographics			
Gender	Female	Male	Male
Age	60	78	67
LVEF at start	30%	20%	20%
Reason for WCD	Non-ischemic	Ischemic	Ischemic
Shocks			
Day of Shock	36	6	17
Pre-shock rhythm	VT	VF	VF
Number of shocks	1	1	1
Survival at 24 h	Yes	Yes	Yes
ICD implanted			
Received ICD	Yes	Yes	Yes

*ICD*, implantable cardioverter-defibrillator; *VF*, ventricular fibrillation; *VT*, ventricular tachycardia; *WCD*, wearable cardioverter-defibrillator

### Remote monitoring arrhythmia detection

During our study, WCD-based remote monitoring of 2794 ECG recordings were available. Arrhythmia monitoring beyond occurrence of VT/VF was reported via automatic downloads of ECG records when non-sinus rhythm was detected. During the 90 days of prescribed wear, new atrial fibrillation was detected in 1 (1%) LVEF ≤ 35% and 3 (7%) LVEF > 35% patients. A total of 2202 recordings in 96 patients (80%) were artifacts (non-VT/VF, non-atrial fibrillation). In total, 60 patients (50%) initiated 443 recordings manually by pressing the WCD response buttons for 3 s. There was no other supraventricular arrhythmia detected.

### ICD implantation

In the absence of VT/VF, the decision to implant an ICD correlated with LVEF at follow-up (Table 1). For example, 68 (89%) patients in the LVEF < 35% group versus 3 (7%) among the LVEF > 35% group were implanted with an ICD for prevention of SCD (*p* < 0.001). There were, however, 5 (4%) patients in the LVEF ≤ 35% group, where the decision to implant was unavailable and corresponded with patient decision to receive follow-up care at another institution.

Subgroup analysis of the reason for WCD prescription along with the decision whether to implant an ICD after 90 days of WCD use indicated that among the LVEF < 35% group, there were 29 out of 29 ICM and 39 out of 44 NICM patients implanted with an ICD. In contrast, among the LVEF > 35% group, there were 15 out of 17 ICM patients and 24 out of 25 NICM patients who were not implanted.

## Discussion

The goal of our analysis was to evaluate whether patients residing in a semirural region and cared for at a community hospital benefitted from wearing the WCD during optimization of GDMT. Our single-center experience with a semirural patient population compared favorably to previously reported outcomes of WCD patients in Germany. The PROLONG cohort reported outcomes from 156 patients enrolled at a single center located in urban Hannover, Germany [12]. They identified LVEF improvement during 90 days of WCD use of 58 (37%) and reported VT/VF events in 11 (7%) patients [12]. These results were similar to the outcomes from the community acute care hospital, in which 44 (37%) patients improved LVEF to > 35% after 90 days and recorded 3 (2.5%) VT/VF events. The PROLONG investigators reported also on a sub-analysis of 117 non-ischemic cardiomyopathy (NICM) patients and identified 6 (5%) in whom VT/VF occurred during WCD use [13].

In an independent publication, the German national experience with the WCD reported that out of 6043 patients, 1.3% of non-ischemic and 1.4% of ischemic patients received an appropriate shock for VT/VF [6]. Similarly, when WCD use among the semirural patient population was examined, VT/VF also occurred among both categories, 1 out of 69 non-ischemic and 2 out of 46 ischemic patients. In the current study, the 3 patients shocked for VT/VF were converted by the first shock; these patients survived long enough to receive an ICD implant and no patient in the current study received an inappropriate shock, not unexpectedly given the 0.4% frequency of inappropriate shocks reported previously in Germany [6]. Our investigation confirmed that improved LVEF > 35% can be achieved by a large number of patients, and agree with the German national experience and the PROLONG study that newly diagnosed cardiomyopathy patients, including those with NICM and LVEF ≤ 35%, were at elevated risk of VT/VF during the period of LVEF recovery [6, 12, 13].

In clinical practice, the recovery process and titration of medication under GDMT cannot be hurried. Use of the WCD allows time to become part of the therapy prior to risk stratification for permanent SCD prevention in patients diagnosed with ischemic as well as non-ischemic cardiomyopathy. After 90 days of GDMT with WCD use, 41 (93%) of the 44 patients with improved LVEF > 35% did not receive an ICD. In contrast, 68 (89%) of the 76 patients with LVEF ≤ 35% at 90 days were ICD implanted. Moreover, one-third of patients improved their LVEF to > 35% independent of the severity of LVEF impairment, age, or gender. Sub-analysis of the semirural patients by starting LVEF ≤ 25% or > 25% indicated that after 90 days of WCD wear, one-third of patients from either group responded to GDMT with improved LVEF > 35%. This illustrates the difficulty of separating patients who will recover from those who will not prior to optimization of GDMT.

Evidence that the WCD patient benefits in ways beyond delivery of shock were also provided by the WEARIT-II study, a prospective registry of 2000 WCD patients from the USA [7]. In addition to detection of VT/VF in 41 (2.1%) patients, the WEARIT-II investigators reported that the WCD recorded non-sustained VT in 28 (1.4%) patients and new atrial arrhythmia in 72 (3.6%). Consistent with WEARIT-II, the current study identified 4 (3.3%) patients with new atrial fibrillation.

In contrast to results reported by observational studies [6–10, 12, 13], the interventional randomized control VEST failed to achieve statistical significance for the primary endpoint of SCD reduction at 90 days [5]. However, interpretation of the primary endpoint is confounded by the likelihood that the study was underpowered due to less than anticipated daily wear of the WCD and the potential to undercount arrhythmic death [5, 14]. When considered along with observational studies, the results of VEST demand a nuanced approach when discussing use of the WCD with the at risk patient.

Although exploratory, as-treated analyses of VEST identified significantly fewer deaths among patients wearing the WCD while indicating that the majority of arrhythmic deaths occurred among patients with VT/VF recurrence [5]. For example, analysis of the 6 deaths that occurred among the WCD group indicated that 5 occurred among those with immediately recurrent VT/VF. In contrast, 14 patients shocked for a single instance of life-threatening arrhythmia survived to the 90 days study endpoint [5]. As a result, patients at risk for SCD may in fact benefit from the WCD, although those experiencing arrhythmic storm are especially in need of timely access to advanced medical care. In the case of the WCD, physicians must continue to weigh the perceived risk of SCD against whether the absence of evidence reported by the VEST investigators is a valid enough justification for inaction [14, 15].

The European Society of Cardiology and the German Cardiology Association have published that the WCD can save lives in patients at risk for VT/VF [16, 17]. Current recommendations for WCD prescription are class IIa–c for use after an ICD explantation, diagnosis of acute myocarditis, and in patients on the heart transplantation waiting list [16, 17]. It is recommended as class IIb–c in a large number of patients with other cardiomyopathies who are expected to achieve improved LVEF (e.g., after coronary artery bypass graft, percutaneous coronary intervention, acute MI, dilated cardiomyopathy, and non-ischemic cardiomyopathy).

In Germany as well as other countries, the WCD is commonly prescribed to patients considered to be at risk for SCD and its efficacy is well documented. As a result, there is ample reason to believe that clinical equipoise will prevent the execution of new randomized control trials requiring that at risk patients are denied access to the WCD. It is common, however, to recommend multiple large-scale randomized control trials to define the treatment of therapy, particularly when reporting community-based studies. Based on our experience, we recommend that lives already saved by appropriate WCD shocks have provided sufficient support for classification of the WCD as a class I standard of care for the patient at risk for sudden cardiac arrest.

### Limitations

The study has several strengths and limitations. This study is the first to describe a cohort of consecutive patients enrolled from a community-based acute care hospital serving a semirural region. Along with the PROLONG study cohort, this study is one of the first to report LVEF at the beginning and end of WCD use. Limitations include the absence of data on common cardiovascular comorbidities. The definitions and hierarchy used to assign patients to a single cardiac diagnosis likely resulted in overlap among disease etiologies. Additional limitations include the lack of a control group of patients who did not receive the WCD. The possibility that the use of a WCD leads to better care, and subsequent improvement in LVEF, may exist. Inherent to the study design of a non-randomized observational analysis is the possibility of selection bias.

## Conclusions

In a community care setting, the impact of WCD use on long-term decision for ICD implantation resulted from identifiable changes in clinical status, such as LVEF improvement or occurrence of VT/VF. Age, gender, and initial LVEF did not predict outcomes. During optimization of GDMT while using the WCD, patient clinical status can improve, resulting in a significantly decreased risk for SCD. Use of the WCD protects patients from SCD until response to GDMT is determined.

## Data Availability

All patient data were collected, statistically analyzed, and archived in the Sana Klinikum Hof, Germany.

## Abbreviations

DCM: Dilative cardiomyopathy
ECG: Electrocardiogram
ESC: European Society of Cardiology
EMS: Emergency medical services
GDMT: Guideline-directed medical therapy
ICD: Implantable cardioverter-defibrillator
ICM: Ischemic cardiomyopathy
IQR: Interquartile range
LVEF: Left ventricular ejection fraction
MI: Myocardial infarction
NICM: Non-ischemic cardiomyopathy
PCI: Percutaneous coronary intervention
SCD: Sudden cardiac death
VEST: Vest Prevention of Early Sudden Death Trial
VF: Ventricular fibrillation
VT: Ventricular tachycardia
WCD: Wearable cardioverter-defibrillator
